# SWeRF—A Method for Estimating the Relevant Fine Particle Fraction in Bulk Materials for Classification and Labelling Purposes

**DOI:** 10.1093/annhyg/met076

**Published:** 2014-01-03

**Authors:** Ingeborg Pensis, Frank Luetzenkirchen, Bernd Friede

**Affiliations:** ^1.^Sibelco Europe MineralsPlus (Ankerpoort NV), Op de Bos 300, NL-6223 EP Maastricht, The Netherlands;; ^2.^Quarzwerke GmbH, Kaskadenweg 40, 50226 Frechen, Germany;; ^3.^Elkem AS, Silicon Materials, PO Box 8126, Vågsbygd, 4675 Kristiansand, Norway

**Keywords:** CLP, crystalline silica, fine fraction of crystalline silica, GHS, industrial minerals, quartz, SWeRF

## Abstract

In accordance with the European regulation for classification, labelling and packaging of substances and mixtures (CLP) as well as the criteria as set out in the Globally Harmonized System (GHS), fine fraction of crystalline silica (CS) has been classified as a specific target organ toxicity, the specific organ in this case being the lung. Generic cut-off values for products containing a fine fraction of CS trigger the need for a method for the quantification of the fine fraction of CS in bulk materials. This article describes the so-called SWeRF method, the size-weighted relevant fine fraction. The SWeRF method combines the particle size distribution of a powder with probability factors from the EN 481 standard and allows the relevant fine fraction of a material to be calculated. The SWeRF method has been validated with a number of industrial minerals. This will enable manufacturers and blenders to apply the CLP and GHS criteria for the classification of mineral products containing RCS a fine fraction of CS.

## INTRODUCTION

The inorganic chemical compound silicon dioxide, also known as silica, exists in many different forms. Quartz is by far the most common crystalline silica polymorph. Quartz is the second most common mineral on the earth’s surface and is found in almost every type of rock, quartz is an important industrial mineral with a multitude of technical applications. It is used in glass, ceramics, foundry castings, filtration, construction, and in its finest flour form is used as a reinforcing filler in paints and plastics, polymer compounds, rubber, sealants, adhesives, etc.

Occupational exposure to respirable crystalline silica, hereafter named RCS, is associated with the lung disease silicosis. The convention that defines the respirable size fraction is EN 481 (CEN, 1993). In the absence of a harmonized classification of crystalline silica in Europe, it has been common practice in the minerals sector for many years to self-classify and label crystalline silica flours as harmful in accordance with the European Dangerous Substances Directive 67/548/EEC (Council Directive, 1967). The label Xn for harmful and the risk phrase R48/20 (danger of serious damage to health by prolonged exposure through inhalation) were assigned to quartz flours.

The European Regulation (EC) No. 1272/2008, also called CLP (EC, 2008), aligns the European Union system of classification, labelling and packaging of substances and mixtures to the UN Globally Harmonized System of Classification and Labelling of Chemicals, GHS (United Nations, 2011). The CLP regulation requires manufacturers, importers, and downstream users to classify substances or mixtures according to the harmonized classification criteria for physical, health, or environmental hazards. CLP Articles 5, 6, and 8.6 clearly point out that available and new information on substances and mixtures ‘shall relate to the form or physical state(s) in which the substance or mixture is placed on the market and in which it can reasonably be expected to be used’. Furthermore, CLP Title V required that by 1 December 2010, substances that met the criteria for classification as hazardous according to the CLP Regulation or substances subject to registration under REACH (Regulation (EC) No 1907/2006 concerning the Registration, Evaluation, Authorisation and Restriction of Chemicals.) must have been notified to the classification and labelling inventory of the European Chemicals Agency.

In accordance with the CLP Regulation, industrial minerals producers have commissioned independent scientific experts to review and reassess the health effects of quartz and cristobalite. The conclusion was that only the fine fraction of CS but not quartz or cristobalite *per se*, fulfils the harmonized criteria for hazard classification. In a group notification to the classification and labelling inventory, classification of the fine fraction of CS as STOT RE Category 1 for the silicosis hazard has been proposed. STOT stands for ‘specific target organ toxicity’ and RE is the acronym for ‘repeated exposure’. The corresponding label is displayed in Fig. 1.

**1:**
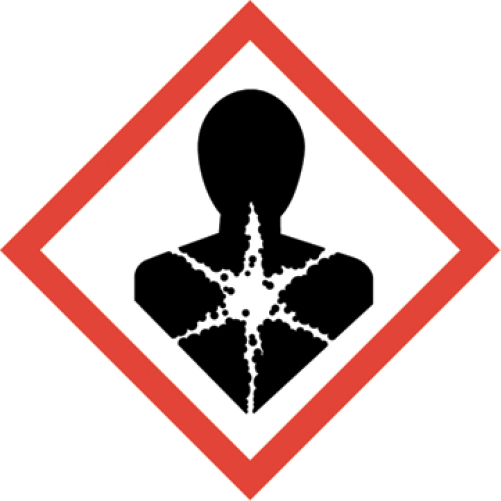
Pictogram for STOT according to the GHS.

The CLP regulation also defines generic concentration limits of ingredients of a mixture classified as a STOT, which trigger classification of the entire mixture. Thus, mixtures and substances containing a fine fraction of CS whether in the form of an identified impurity, additive, or individual constituent, will be classified as STOT RE 1, if the CS (fine fraction) concentration is ≥10% w/w. They will be classified as STOT RE 2, if the CS (fine fraction) concentration is between 1 and 10% w/w. If the CS (fine fraction) content in mixtures or substances is below 1%, no hazard classification of the product is required.

In order to classify a product containing a fine fraction of CS under the CLP regulation, it is necessary for manufacturers of such products to quantify the relevant fine fraction of CS of their respective products. However, there is currently no validated method available for the quantification of the relevant fine fraction of CS in bulk materials. In occupational hygiene, the term respirable implies that a particle is actually airborne, and it seems illogical to measure airborne particles in a bulk material. The solution to this dilemma is to define the relevant fine fraction of CS in bulk materials as crystalline silica particles that, if made airborne and subsequently inhaled, can potentially penetrate to the lungs’ unciliated airways. Based on this approach, the Metrology Working Group of the European Industrial Minerals Association, IMA-Europe, has developed the principle of the size-weighted relevant fine fraction (SWeRF) for estimating the potential respirable fraction in bulk materials for classification and labelling purposes. The principle behind SWeRF is to combine the size of a particle with its probability to enter the lungs’ unciliated airways as described in the EN 481 standard.

## METHODS

When dust is inhaled, smaller particles have a higher probability to reach the alveoli in the lungs than larger particles. The function that describes the relationship between the size of a particle and this probability is specified in the EN 481 standard. For example, particles with an aerodynamic diameter of 1 or 10 µm have, according to EN 481, a probability to reach the alveoli of 97.1 and 1.3%, respectively. The principle behind SWeRF is to combine the particle size with the above-mentioned probability factor and to integrate this for the whole size distribution range of a material. The SWeRF is the fraction of a material in which the contribution of each particle is weighted as a function of its size. SWeRF is thus a method to predict how particles in a bulk material would move when they become airborne and are inhaled. There are in principle two ways to determine the SWeRF of a material. One is to first determine the particle size distribution (PSD) of the material and then calculate the SWeRF from this PSD using the probability function of EN 481. The other way is dispersing the material in a liquid in which the SWeRF is separated by separating the larger particles from the smaller particles by sedimentation.

### Calculation of SWeRF from PSD

The starting point of the SWeRF calculation is the known PSD of the respective sample, e.g. derived from laser diffraction. The experimental conditions for the PSD measurement will depend on the material to be investigated. Parameters that need to be considered are dispersing agents, dispersing media, ultrasonic energy, concentration, optical settings, and calculation model, i.e. Fraunhofer versus Mie theory (ISO, 2007, 2009). It is important to point out at this stage that the spherical equivalent diameter, which is measured by laser diffraction techniques, is not equal to the aerodynamic diameter as referred to in EN 481. The relation between the two diameters is given in equation (1).


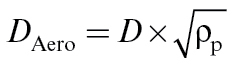
(1)

A size weighting is then applied, based on the probability function given in EN 481, i.e. the probability function for particles reaching the alveoli when inhaled.


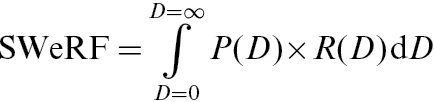
(2)

where *P*(*D*) is the particle size fraction for aerodynamic diameter D; *R*(*D*) the probability of reaching the alveoli for particles of aerodynamic diameter *D*, according to EN 481; and *D* is the aerodynamic diameter = *d* × √(SG), where SG is the specific gravity.

As a courtesy and in order to facilitate the use of SWeRF, a publicly available SWeRF calculation spreadsheet has been provided (IMA-Europe, 2013a).

### Measuring the SWeRF by sedimentation

In the case that crystalline silica particles do not have the same size distribution as the other particles in the material or that the particle shape is different, it is possible to separate the SWeRF by sedimentation. In the same way as fine particles have a higher probability to enter deep into the lungs, they also have a higher probability to remain in suspension, which is expressed by equation (3).

Both the respirable convention and the separation by sedimentation are probability functions. *R*(*D*) describes the probability for a particle to enter the alveoli (EN 481) and *S*(*D*) describes the probability that a particle remains in suspension. The SWeRF is found by choosing the parameters for sedimentation in such a way that the sum of all probabilities for all diameters is equal for both the respirable convention and the sedimentation. This is done by equating the integrals of both functions.


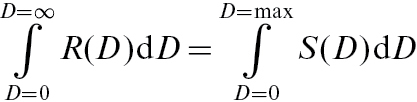
(3)

where *R*(*D*) is the probability for a particle to enter the alveoli (EN 481) and *S*(*D*) is the probability that a particle remains in suspension.

The SWeRF is found by choosing the parameters for sedimentation in such a way that the sum of all probabilities for each diameter is equal for both the respirable convention and the sedimentation. This is done by equating the integrals of both functions.

The time of sedimentation can be calculated using equation (4), which is based on Stokes’ law and the convention described in EN 481.


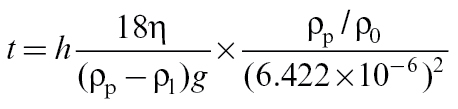
(4)

where *t* is the time (s) at which separation of the particles by sedimentation in the liquid equals separation according to EN 481, *h* the height (m) of the column of the supernatant liquid that is extracted after time = *t*, η the dynamic viscosity of the liquid (kg ms^−1^), *g* the acceleration due to gravity (m s^−2^), ρ_p_ the density of the solid particles (kg m^−3^), ρ_0_ the unit density (kg m^−3^), and ρ_l_ is the density of the liquid (kg m^−3^).

A publicly available document describing the derivation of the equation (3) has been provided by IMA-Europe (2013b).

For determining the SWeRF of a sample, the density of this sample should be used for ρ_p_. When the SWeRF of crystalline silica, SWeRF_CS_, is determined, the density of crystalline silica should be used.

The SWeRF of the sample can then be determined using equation (5).


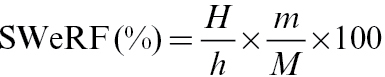
(5)

where *H* is the height of the total column of fluid that is used for sedimentation, *h* the height to which the supernatant is extracted at the calculated time, *M* the total mass that was dispersed, and *m* is the mass of the residue in the extracted supernatant.

For a pure quartz or cristobalite sample, the SWeRF gives directly the relative fine fraction of CS. For a mixture, the relative fine fraction of CS of the sample, SWeRF_CS_, can be calculated by using equation (6).


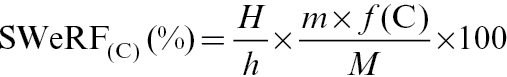
(6)

where SWeRF(_C_) is the SWeRF of constituent C and *f*(C) is the fraction of constituent C in the residue in the supernatant.

### Experimental sedimentation procedure

A suitable sedimentation liquid must meet the following requirements: the particles in the sample must be completely de-agglomerated, should not dissolve, swell or disintegrate, and should not react with the liquid. For many minerals, water is a suitable sedimentation liquid. For materials like cement, a non-aqueous liquid, e.g. alcohol, should be considered.

The sample is dispersed in a suitable liquid. After the calculated sedimentation time, the supernatant of the dispersed sample is analysed for the respirable content, i.e. total solids within the supernatant, and crystalline silica content.

The mineral sample is weighed (*M*, mg) and dispersed in 50ml of the liquid in a 100ml glass beaker. The volume of the solid should be maximum 1% of the volume of the total liquid to ensure unhindered sedimentation of the individual particles. A typical value is 5g. To achieve optimum dispersion and de-agglomeration, ultrasound should be applied. To prevent the sample from flocculating or coagulating, addition of a suitable dispersant, such as polyphosphate, might be required. The dispersed sample is then transferred to a 250ml glass cylinder, filled up with liquid to 250ml, and homogenized. The cylinder is then left to settle for the calculated time without agitation or vibration. The height of the liquid column *H* (mm) is recorded. After the calculated settling time, the supernatant *h* (mm) is collected with a pipette. After evaporation of the supernatant, the remaining weight of suspended material *m* (mg) is measured. The SWeRF of the sample can be calculated using equation (5). The content of crystalline silica of this separated material can be determined using X-ray diffraction or infrared techniques. The SWeRF_CS_ of the sample is calculated using equation (6). It should be noted that for determining the SWeRF of a material, the density of this material should be used to calculate the time of separation by sedimentation. If the SWeRF_CS_ is required, the density of quartz or cristobalite, respectively, should be used (Fig. 2).

**2:**
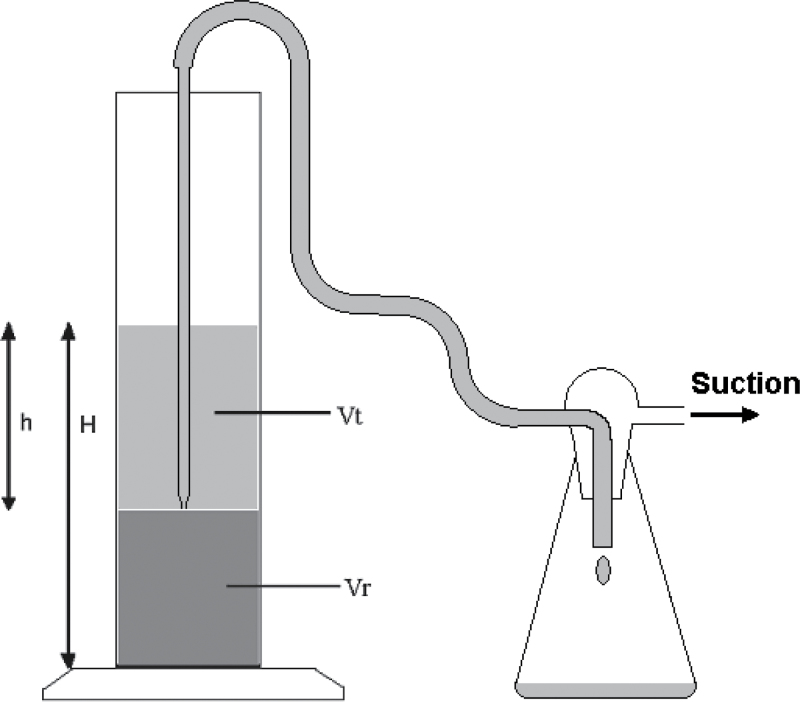
Experimental set-up of the sedimentation.

## RESULTS

### Quartz samples

The SWeRF of eight commercial quartz products, with grain sizes ranging from coarse sand to fine flour, supplied by Quarzwerke and SCR-Sibelco, was determined both by calculation from the PSD and gravimetrically by the sedimentation technique. The *D*_50_ values of the quartz samples and the SWeRF results are listed in Table 1. The correlation between the different techniques is displayed in Fig. 3.

**Table 1.** Comparative study of the SWeRF of commercial quartz products, determined by calculation and sedimentation

Sample no.	*D*_50_ (µm)	SWeRF calculated from PSD	SWeRF sedimentation
Quartz 4	150.0	0.0	0.0
Quartz 3	90.0	3.2	2.4
Quartz 5	17.4	11.9	13.2
Quartz 6	9.9	18.5	18.0
Quartz 7	7.2	22.3	22.1
Quartz 8	3.2	38.3	39.8
Quartz 2	3.0	42.2	38.3
Quartz 1	1.3	52.5	59.4

**3:**
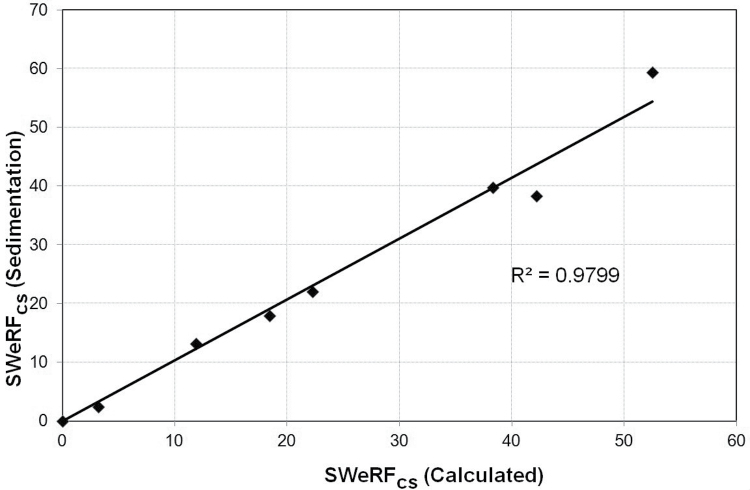
Correlation between calculation and sedimentation methods for the SWeRF of commercial quartz samples.

### Spiking of barite with quartz

The measurement of the SWeRF of pure quartz samples is straightforward as there are no interferences with other phases. Due to its ubiquitous nature, traces of quartz are present in many other minerals. In order to investigate whether the SWeRF_CS_ of a quartz-containing mineral blend can be determined, a quartz-free pure barite sample (BaSO_4_) from Ankerpoort NV was spiked with different amounts of quartz flour (supplied by Quarzwerke). The SWeRF of the quartz flour was calculated from its PSD, giving a SWeRF of 41.4%. Analysis of the chemical composition of the mineral blends by means of X-ray fluorescence confirmed the homogeneity of the samples. The SWeRF_CS_ of the quartz-spiked barite samples was calculated based on the known SWeRF of the quartz flour and also determined by sedimentation technique. The details and results are given in Table 2. The correlation between the expected calculated SWeRF and the experimentally determined SWeRF is shown in Fig. 4. The *R* ^2^ value of 99.3% is a measurand for the recovery of the spiking material in the mineral blend.

**Table 2.** Determination of SWeRF in quartz-spiked barite samples

Percentage of quartz added	Calculated from EN 481	Sedimentation method		
	SWeRF_CS_	SWeRF	Percentage of quartz in SWeRF (FT-IR)	SWeRF_CS_
0	0	0	0	0
25	11.3	34.5	28.3	9.8
50	21.9	38.1	58.3	22.2
75	32.0	40.8	79.4	32.4
100	41.4	41.8	91.9	38.4

FT-IR = Fourier transform infrared spectroscopy.

**4:**
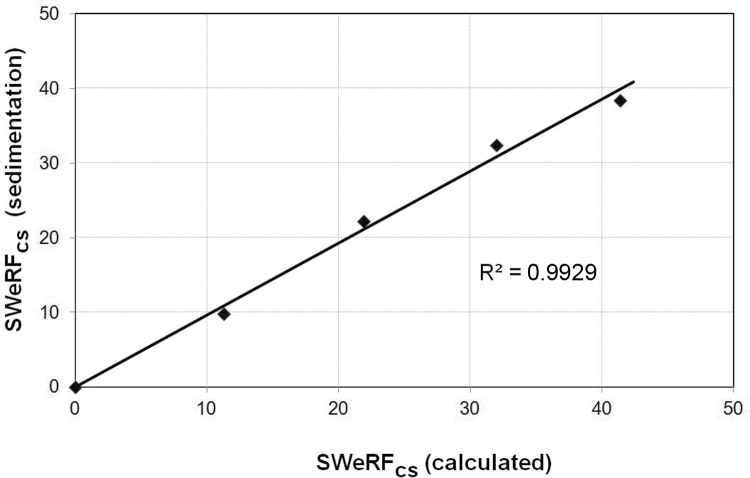
Correlation between expected (calculated) and measured (sedimentation) SWeRF of quartz-spiked barite.

### Industrial minerals

The SWeRF method has been applied to a variety of widely used commercial industrial mineral products, supplied by a number of European producers. The list of industrial minerals comprises calcium carbonate, feldspar, kaolin, bentonite, mica, wollastonite, sepiolite, talc, diatomaceous earth, cristobalite, clay, lime, iron ore, chromite ore, manganese ore, and zeolites. Because of the multitude of industrial mineral products tested and their large variability regarding granulometry and quartz content, only the results of diatomaceous earth products are presented in this article. The results are displayed in Table 3.

**Table 3.** Determination of SWeRF in diatomaceous earth products

	Flux calcined				Calcined
	Very fine	Very fine	Fine	Fine	Coarse
*D*_50_ (µm)	9.1	9.3	13.8	17.4	21.5
SWeRF sedimentation (%)	16.5	15.4	6.9	4.7	6.7
SWeRF calculated (%)	19.4	17.0	8.5	4.9	8.0

The correlation between sedimentation technique and calculation is very good (*R* ^2^ = 0.9915). Also the repeatability of the SWeRF sedimentation method gives satisfying results. Table 4 shows the results of seven measurements of a very fine flux-calcined diatomaceous earth sample with a *D*_50_ of 9.3 µm.

**Table 4.** Repeatability measurements of a flux-calcined diatomaceous earth sample (*D*_50_ = 9.3 µm) by sedimentation technique

Test no.	SWeRF sedimentation (%)
1	17.6
2	14.9
3	14.8
4	17.1
5	14.9
6	13.6
7	15.2
Mean	15.4
Standard deviation	1.41

### Round robin test

A round robin test was carried out to validate the SWeRF method (Quarzwerke, unpublished results). Twenty-two members of IMA-Europe participated with measurements in one or more laboratories using various instruments for PSD measurements and sedimentation. Results were provided for laser diffraction (30 laboratories), sedimentation with X-ray absorption measurement (12 laboratories), and SWeRF sedimentation analysis (13 laboratories). The test material was pure quartz flour produced from processed silica sand of the Frechen deposit by iron-free grinding and subsequent air separation (Quarzwerke, Germany). Since optical settings in laser diffraction strongly influence the results, the laboratories agreed on the following harmonized parameters for the analysis according to the Mie theory: refractive index of quartz (1.54), absorption coefficient of quartz (0.1), and refractive index of water (1.33). The results are displayed in Fig. 5. Results from laboratories that did not carry out the analyses according to the test protocol were discarded.

**5:**
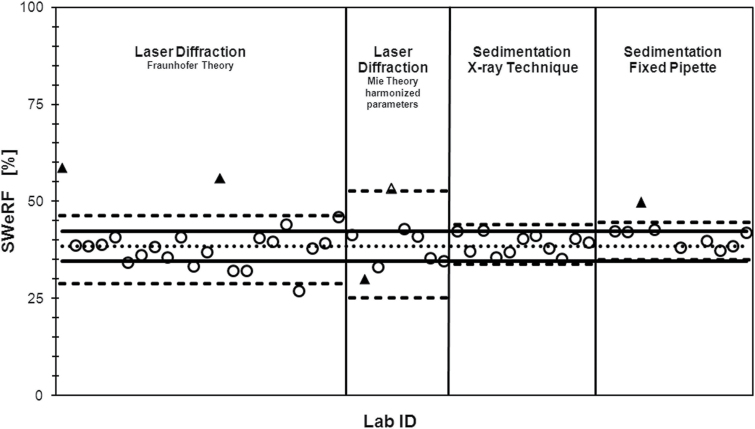
Reproducibility of SWeRF measurements with different techniques. Dotted line: target value; dashed line: statistic tolerance limits of the method; solid line: tolerance around mean value with 10% standard deviation; and triangles: statistical outliers according to Dixon (DIN 53804-1; Deutsches Institut für Normung e.V. (German National Standard), 2002).

## DISCUSSION

The tested quartz products showed a good correlation between the calculated SWeRF_CS_ and the SWeRF_CS_ determined by sedimentation. According to the specific concentration limits as laid down in the CLP regulation, the fine quartz flours with a relevant fine fraction of CS above 10% fulfil the classification criteria as STOT RE 1, whereas coarse quartz flours with an a relevant fine fraction of CS between 1 and 10% fulfil the classification criteria as STOT RE 2. Quartz sand with a relevant fine fraction of CS content below the generic cut-off value of 1% does not require to be hazard classified and labelled.

The spiking experiment of quartz flour in barite showed that quartz could be recovered proportionally from a mixture containing a mineral of higher specific gravity, which is 4.48 for barite and 2.66 for quartz, respectively. Spiking showed that differences in density have no impact on the determination of SWeRF_CS_. The correlation between theoretical values and measured values from SWeRF sedimentation was excellent and resulted in an *R* ^2^ value of 99.3%. The results demonstrate that the SWeRF method can be used to quantify the relevant fine fraction of quartz in mineral mixtures and thus allow classification and labelling according to the CLP regulation.

The spiking of high purity barite with known amounts of quartz flour is of course an ideal case. In cases where quartz particles in the respirable size fraction are incorporated in the mineral matrix, the real fine fraction of CS of the sample might be underestimated. However, the SWeRF still gives the true value for the total relevant fine fraction and the SWeRF_CS_ the quartz content therein. The fine fraction of CS that is not collected in the SWeRF is not relevant for classification and labelling either because it is neither accessible nor respirable. In cases where the mineral is either hydrophobic or floats on water, e.g. cenospheres or surface-treated powders, the SWeRF method has its limitations and either alternative techniques must be used or the method must be adapted.

It thus appears clear that a good knowledge of the physico-chemical properties of the material is an important pre-condition when determining SWeRF. Furthermore, suitable dispersing media must be chosen in order to give reliable results for both laser diffraction and sedimentation. Alternative solvents or additives must be considered in cases where water interacts with the sample. Typical examples are swelling effects in the case of bentonites, chemical reactions in the case of lime, or disintegration and flocculation in the case of clay minerals.

The calculated SWeRF_CS_ of a mineral product is based on the principle that the crystalline silica has the same size distribution as the other minerals in the product. Because of the high hardness and absence of cleavages, quartz is tougher than most minerals and will most likely be rather more present in the coarser fraction. This would result in an overestimation of the calculated SWeRF_CS_. A series of tests with different industrial minerals was performed to verify this.

The outcome of applying the SWeRF method to a variety of industrial minerals was very positive, showing that the size-weighted relevant fine fraction can be determined for all sorts of minerals. For industrial minerals with platy particle shape however, such as kaolin, mica, or talc, adaptations to the method were required, i.e. use of a suitable dispersant, in order to optimize the sedimentation. In the case of porous or hollow particles, the effective density, which is expressed as a combination of skeleton density measured by Helium pycnometry, and the internal porous volume measured by Mercury intrusion porosimetry, must be used when calculating the SWeRF.

The subdivided round robin samples passed the homogeneity check in accordance with ISO 13528 (ISO, 2005). Although the SWeRF results showed some scatter depending on the method, the mean value was at an acceptable level (36–39%). Harmonized parameters like refractive index, absorption coefficient, and the medium refractive index must be considered when applying the Mie theory. The analysis of the laser diffraction data according to the Fraunhofer approximation does not require harmonized optical parameters and is thus the preferred method for samples of mineral blends with unknown or variable optical properties. Four out of 13 results from the sedimentation technique have been omitted from statistical evaluation because the analyses were not carried out in accordance with the test protocol. This underlines the importance of following the standard operating procedure as set out in this article.

The benefit of the SWeRF method is primarily to be able to fulfil classification and labelling requirements of minerals and products that contain a fine fraction of CS. In the light of GHS being implemented in more and more countries all over the world, the SWeRF method has also relevance for classification and labelling (C&L) requirements in countries outside Europe since it quantifies the potential relevant fine fraction of CS in a product. This is relevant for GHS, CLP, or any other regulation that requires quantification.

Safety data sheets (SDS) are effective and well-accepted tools to communicate safety information of chemical products in the supply chain. They have been made an integral part of chemical legislations worldwide. Correct classification and labelling allows both corporate occupational hygienists and downstream users to assess the risk of handling certain products at workplaces, because both pictograms and the SDS text provide important information on intrinsic properties and potential hazards. The SWeRF method thus contributes to assessment of the potential hazard of products containing a fine fraction of CS in SDS and allows—for example—development of less hazardous products or even substitution of products containing large amounts of fine CS.

From a downstream user’s perspective, hazard classification of a product clearly provides important information. Although coarse quartz sand can release hazardous CS dust during handling and use (e.g. sand blasting), the product will still not be hazard classified when the content of the fine fraction of CS is <1%. One has to bear in mind that the classification criteria in GHS and CLP are hazard based and not risk based. There is no hazard phrase that would allow addressing liberation of hazardous dust upon handling. The risk factor related to the generation of hazardous CS dust from handling and use of a specific product is dealt with in section 8 of formal SDS where occupational exposure limits are listed. As long as the global chemical legislations do not cover a dustiness endpoint, there is the need for a method for the quantification of fine fraction of CS in bulk materials. The SWeRF method provides the right tool to address this issue.

The main focus of exposure to RCS is in the workplace atmosphere. Hence, the question often arises whether and how the results of SWeRF measurements correlate to real exposure to RCS in workplaces or to dustiness measurements of powder products.

The purpose of SWeRF is, however, not to predict the levels of exposure. It is a measure of how much CS in the relevant fine fraction is contained in a bulk material in order to fulfil C&L requirements for producers, importers, traders, and compounders. SWeRF is not designed to substitute workplace air measurements. Concentrations of RCS in the air when handling such a material depend on too many factors and cannot be calculated based on SWeRF or dustiness measurements.

Industrial handling of bulk materials, i.e. storage, filling, conveying, and mixing, often leads to dust generation. This represents potential physical hazards with regards to fire and explosions, health hazards related to the inhalation of dusts, as well as contamination of equipment and environmental pollution (Schneider, 2008). The European standard EN 15051 (CEN, 2006) describes the measurement of the dustiness of a powder by using a rotating drum or continuous drop method. The dustiness of a powder product, defined as the propensity of a material to generate airborne dust during its handling (Lidén, 2006), depends on the intrinsic properties of the material and the handling scenario. Measuring the dustiness index of a material can help to assess and reduce dust exposure at workplace as well as to develop less dusty products (Pensis *et al.*, 2010). During dustiness measurements, the respirable fraction of the released dust can be collected on suitable filters and analysed for its RCS content. This allows an estimation of RCS generation during defined handling operations. However, although dustiness measurements provide useful information to occupational hygienists on material properties, dustiness is not an endpoint under GHS and CLP and cannot be used for classification and labelling purposes.

Furthermore, studies comparing dustiness tests with corresponding workplace exposure data have revealed that dustiness can explain ca. 70% of the variance of exposure (Brouwer *et al.*, 2006). However, the success of such work is only limited. Since it has been shown that there is not a consistent relationship between dustiness tests and real workplace exposure measurements, dustiness measurements cannot substitute workplace exposure measurements or even occupational exposure limits (Lidén, 2006). It is also clearly pointed out in the revised EN 15051 standard that dustiness tests are designed for product characterization and not for workplace exposure estimation. There will always be the need to measure real workplace exposure during dust-generating operations, also in order to verify whether dust-reducing properties obtained from dustiness tests or reduced RCS contents from SWeRF measurements for a specific product really are effective.

## CONCLUSIONS

SWeRF is a valid method for the determination of the relevant fine fraction in bulk materials in general and of the relevant fine fraction of CS in particular for classification and labelling purposes in accordance with the CLP regulation. The method has been tested on a multitude of different industrial minerals. Spiking experiments prove the high accuracy of the SWeRF method, whereas a round robin test with 22 participants and 4 different methods proved the reproducibility.

For pure quartz samples, calculation of the SWeRF from granulometry data can be accepted as a method to quantify the relevant fine fraction of CS in a bulk material and hence to determine the correct hazard classification of the product. For other minerals, it must be decided on case-by-case basis whether the sedimentation technique or the calculation from granulometry data is more suitable. In cases of platy mineral shape or porous materials, adaptations to the method might be indicated, e.g. addition of flocculants or dispersants, or the use of effective density rather than specific gravity.

Calculated SWeRF values obtained from granulometry data are influenced by the optical parameters used during laser diffraction measurements, i.e. Fraunhofer versus Mie theory.

The SWeRF method alone is not a suitable method for risk assessment, and it is not meant to be either. SWeRF is not a substitute for workplace exposure measurements. The main purpose is to determine the potential respirable fraction for classification and labelling purposes. However, SWeRF can be a complementary method for workplace atmospheres when combined with standardized measurements of dustiness in accordance with EN 15051.

The SWeRF method has been submitted to the European committee for standardization (CEN).
